# Data Resource Profile: The Japan COVID-19 and Society Internet Survey (JACSIS)

**DOI:** 10.1093/ije/dyag025

**Published:** 2026-03-10

**Authors:** Hidehiro Someko, Keisuke Anan, Takahiro Tabuchi, Takashi Yoshioka, Ryo Okubo, Yuki Furuse, Kota Katanoda, Takeo Fujiwara, Naoki Kondo, Yosuke Yamamoto

**Affiliations:** Department of Healthcare Epidemiology, Graduate School of Medicine, Kyoto University, Kyoto, Japan; Department of Internal Medicine, Nagoya Tokushukai General Hospital, Kasugai, Japan; SRWS-PSG, Osaka, Japan; Department of Healthcare Epidemiology, Graduate School of Medicine, Kyoto University, Kyoto, Japan; SRWS-PSG, Osaka, Japan; Division of Respiratory Medicine, Saiseikai Kumamoto Hospital, Kumamoto, Japan; Division of Epidemiology, School of Public Health, Tohoku University Graduate School of Medicine, Sendai, Japan; Health Technology Assessment Unit, Department of Preventive Medicine and Public Health, Keio University School of Medicine, Tokyo, Japan; Institute of Clinical Epidemiology, Showa University, Tokyo, Japan; Department of Psychiatry, Hokkaido University Graduate School of Medicine, Hokkaido, Japan; Department of Microbiology and Infection, The University of Tokyo Pandemic Preparedness, Infection and Advanced Research Center (UTOPIA), Tokyo, Japan; Department of Medical Virology, Nagasaki University Graduate School of Biomedical Sciences, Nagasaki, Japan; Division of Population Data Science, National Cancer Center Institute for Cancer Control, Tokyo, Japan; Department of Public Health, Institute of Science Tokyo, Tokyo, Japan; Department of Social Epidemiology, Graduate School of Medicine and School of Public Health, Kyoto University, Kyoto, Japan; Department of Healthcare Epidemiology, Graduate School of Medicine, Kyoto University, Kyoto, Japan

**Keywords:** health inequities, repeated survey waves, social determinants of health, web-based panel

## Data resource basics

Key Features

- The Japan COVID-19 and Society Internet Survey (JACSIS) was established in 2020 to investigate the multifaceted impacts of the COVID-19 pandemic, enabling the comparison of pre- and post-pandemic data through linkage with the previous Japan “Society and New Tobacco” Internet Survey (JASTIS) studies (6494 participants from pre-pandemic period; 7351 from pandemic onset).
- The study commenced in August 2020 in Japan with 28 000 initial participants (25 482 after excluding satisficers) comprising Japanese adults aged 16–79 years in Wave 1, expanded to include those aged ≥80 years in Wave 8, with approximately equal gender distribution.
- Follow-up surveys have been conducted twice a year, maintaining ∼30 000 participants per wave through replacement sampling (response rates: ∼60% for previous participants, ∼30% for new panelists); the dataset includes 74 003 unique individuals due to the re-recruitment of previous participants.
- Data collection encompasses demographics, socioeconomic factors, validated health scales such as Kessler-6, COVID-19 assessments (fear of COVID-19, vaccine attitudes, etc.), lifestyle behaviors (tobacco/alcohol use, gambling), social measures (Lubben Scale, childhood experiences), and pandemic impacts on healthcare utilization and daily activities (hospital visits, influenza vaccination, work from home).
- The dataset is available to external researchers through collaborative research frameworks; contact the corresponding author at tabuchitak@gmail.com for the application procedures.

To address critical research needs about the societal impacts of COVID-19, we established the Japan COVID-19 and Society Internet Survey (JACSIS) cohort in August 2020. This internet-based study aims to investigate the multifaceted impacts of the COVID-19 pandemic through four primary objectives: (i) conducting rigorous, data-driven scientific analyses of how the pandemic has affected socioeconomic factors, health outcomes, and overall quality of life across diverse population segments; (ii) disseminating accurate and timely information to the public; (iii) supporting evidence-based policymaking; (iv) contributing to the global body of evidence on COVID-19. A key strength of JACSIS is its ability to link data with the pre-pandemic Japan “Society and New Tobacco” Internet Survey (JASTIS) studies conducted between 2015 and 2020 [1]. The study is funded by the Japan Society for the Promotion of Science (JSPS), Health Labour Sciences Research Grants, Japan Agency for Medical Research and Development (AMED), Japan Science and Technology Agency (JST), and various other governmental and institutional sources.

Participant recruitment was conducted through the commercial research agency Rakuten Insight, whose panel comprises ∼2.2 million individuals from the Japanese population. The cohort consists of Japanese adults aged 16–79 years in Wave 1, expanded to include those aged ≥80 years in Wave 8, with approximately equal gender distribution.

The JACSIS cohort participants were followed through questionnaire surveys conducted twice a year from August 2020. As of February 2024, eight waves have been completed. Survey waves were distributed as follows: Wave 1 (August and September 2020), Wave 3 (September and October 2021), Wave 5 (September and October 2022), and Wave 7 (September–November 2023), while participants were also invited to participate in JASTIS surveys conducted in Wave 2 (February 2021), Wave 4 (February 2022), Wave 6 (February 2023), and Wave 8 (January and February 2024). Each wave maintained ∼30 000 participants through replacement sampling, with retention rates varying from 65% to 81% between consecutive waves and the cumulative retention from Wave 1 declining to 24% by Wave 8 (Table 1). However, the seemingly low retention rate reflects participants who responded to every single wave consecutively. Our recruitment strategy allows previous participants to rejoin after missing waves, resulting in 11 749 participants providing valid responses to both Wave 1 and Wave 8, and a total of 74 003 unique individuals participating across all waves. The response rates were ∼60% for participants from previous waves and 5%–10% for newly recruited panelists from Rakuten Insight. Despite the dynamic nature of the cohort composition across the waves, the distribution of demographic variables and socioeconomic statuses in our cohort is similar to those of nationally representative samples (Table 2 and Supplementary Table S1).

**Table 1:** Number of eligible responses, response rate, dropout, and follow-up rate of each wave.

Wave	Timing	Participants	Eligible responses	Dropouts from previous waves	Newly added	Retained participants from previous waves [*N* (%)]^a^	Participants retained consecutively from Wave 1 [*N* (%)]^a^	Response rate of participants from previous waves including JASTIS 2015–2020 (%)	Response rate of new participants from Rakuten Insight panelist (%)
Wave 1	Aug–Sep 2020	28 000	25 482	–	–	–	–	–	–
Wave 2	Feb 2021	26.000	23 142	5160	3160	18 803 (74)	18 803 (74)	73.3	18.7
Wave 3	Sep–Oct 2021	31 000	28 175	6660	11 660	15 976 (69)	14 370 (56)	69.0	31.2
Wave 4	Feb 2022	33 000	30 130	4134	6134	22 756 (81)	12 204 (48)	71.9	2.6
Wave 5	Sep–Oct 2022	32 000	28 630	7384	6384	21 396 (71)	9909 (39)	65.7	17.7
Wave 6	Feb 2023	34 000	31 037	5789	7789	21 976 (77)	8486 (33)	63.6	5.5
Wave 7	Sep–Nov 2023	33 000	28 481	9341	8341	20 052 (65)	6808 (27)	57.4	10.8
Wave 8	Jan–Feb 2024	32 000	29 268	5125	4125	22 663 (80)	6065 (24)	62.9	–^b^

a Number of retained participants are calculated only among participants who provided eligible responses in each wave. The retention rate represents the proportion of participants who provided eligible responses in both the previous wave and the current wave. The sum of retained and newly added participants does not equal the total number of eligible responses due to participants who did not provide eligible responses in previous waves but did so in the current wave.

b We did not recruit new participants from Rakuten Insight panelists because we fulfilled the target sample size by recruiting participants from previous waves including the Japan Society and New Tobacco Internet Survey 2015–2020. The recruitment period was similar to those of other waves.

**Table 2:** Characteristics of the cohorts in Wave 1 and Wave 8, compared with the Japanese population.

	Wave 1		Wave 8		% of Japanese population^b^
	**Unweighted**	**Weighted**	**Unweighted**	**Weighted**	
**Characteristic**	***N* = 25 482** ^a^	***N* = 25 482** ^a^	***N* = 29 268** ^a^	***N* = 29 268** ^a^	
Age group (years)					
0–15	0 (0)	0 (0)	0 (0)	0 (0)	12.1
16–19	1214 (4.8)	1214 (4.8)	521 (1.8)	521 (1.8)	4.5
20–29	3211 (13)	3211 (13)	5043 (17)	5043 (17)	10.4
30–39	3767 (15)	3767 (15)	4825 (16)	4825 (16)	11.6
40–49	4894 (19)	4894 (19)	5037 (17)	5037 (17)	14.8
50–59	4256 (17)	4256 (17)	4621 (16)	4621 (16)	13.2
60–69	4243 (17)	4243 (17)	4485 (15)	4485 (15)	11.1
70–79	3897 (15)	3897 (15)	4123 (14)	3999 (14)	11.7
≥80	0 (0)	0 (0)	613 (2.1)	737 (2.5)	7.8
Sex					
Female	12 809 (50)	12 809 (50)	14 816 (51)	14 816 (51)	48.7
Academic attainment					
Junior-high-school graduate	1014 (4.0)	2606 (10)	541 (1.8)	1419 (4.8)	13.1
High-school graduate	8375 (33)	9969 (39)	7554 (26)	14 385 (49)	44.2
College graduate	5259 (21)	4559 (18)	6114 (21)	5572 (19)	16.2
Bachelor’s degree	9705 (38)	5915 (23)	13 319 (46)	6628 (23)	23.1
Master’s or doctoral degree	1067 (4.2)	2226 (8.7)	1459 (5.0)	670 (2.3)	2.4
Other	62 (0.2)	207 (0.8)	281 (1.0)	595 (2.0)	0
Marital status					
Married	15 230 (60)	16 100 (63)	17 194 (59)	18 144 (62)	58.3
Never married	7806 (31)	6046 (24)	9356 (32)	8174 (28)	27.4
Widowed or divorced	2446 (9.6)	3336 (13)	2718 (9.3)	2951 (10)	14.2
Household equivalent income^c^					
Low	2352 (9.2)	2282 (9.0)	2830 (9.7)	3064 (10)	19
Medium	5260 (21)	5377 (21)	5721 (20)	5670 (19)	26.4
High	12 596 (49)	12 228 (48)	14 214 (49)	13 247 (45)	54.6
Unknown/declined to answer	5274 (21)	5595 (22)	6503 (22)	7287 (25)	0
Type of employment					
Employer	847 (3.3)	1008 (4.0)	988 (3.4)	959 (3.3)	2.8
Self-employed	1449 (5.7)	1667 (6.5)	1152 (3.9)	1238 (4.2)	6.1
Regular employee	8666 (34)	7876 (31)	11 146 (38)	10 487 (36)	30.1
Non-regular employee	4492 (18)	5210 (20)	6666 (23)	6769 (23)	19.1
Unemployed	10 028 (39)	9722 (38)	9316 (32)	9815 (34)	41.1

a Median (Q1, Q3); *n* (%).

b Data of Japanese population came from National census 2020 (age, sex) and Comprehensive Survey of Living Conditions 2019 (academic attainment, marital status, household equivalent income, type of employment).

c Low indicates income 0–200 million Japanese Yen (JPY), middle 200–400 million JPY, high >400 million JPY.

The JACSIS cohort has secured research funding through to 2028, ensuring the continuation of data collection at least through to Wave 13. Wave 9 has already been completed and is currently in the data-cleaning phase, while preparations for Wave 10 are underway, with the research team finalizing the survey items. The investigators anticipate securing additional funding for 2029 and beyond.

For participants aged 16–17 years, informed consent was obtained directly from them without parental consent in accordance with the Ethical Guidelines for Medical and Biological Research Involving Human Subjects implemented by Japan’s Ministry of Education, Culture, Sports, Science and Technology and Japan’s Ministry of Health, Labour and Welfare [2]. Ethics approval information is provided below.

## Data collected

The JACSIS dataset is a primary data-collection initiative using a repeat panel design. Data collection was conducted via Rakuten Insight’s secure web platform, with participants receiving compensation through a credit point system known as “E-points,” which could be used for internet shopping and cash conversion.

In determining the cohort size, we established a final target sample size of 28 000 participants in Wave 1. This figure was determined based on budgetary constraints while ensuring adequate sample sizes within each sex and age stratum for reliable statistical analyses, including propensity score modeling and event-rate estimation. As a multipurpose study designed to address diverse research questions, formal power calculations for specific hypotheses were not conducted.

The JACSIS dataset is produced through internet-based questionnaires administered to participants from Rakuten Insight’s panel of ∼2.2 million Japanese individuals. For initial recruitment in Wave 1, a stratified sampling strategy was employed based on sex, age, and geographic region to match the intended representative distribution. After conducting a preliminary response rate assessment with 28 000 randomly selected panelists, Rakuten Insight adjusted the number of invitations to achieve the target sample size, resulting in a final participation rate of 12.5% (28 000/224 389). For subsequent waves, our recruitment strategy has followed a two-step process. First, we invite all individuals who have participated in any previous wave (including JASTIS 2015–2019) to rejoin the study. If this approach does not yield sufficient participants to meet our target sample size (which may vary slightly based on budget constraints and other factors), we then recruit new participants from Rakuten Insight’s panel by using the same stratified sampling method as employed in Wave 1 (stratified by sex, age, and geographic region). Therefore, while a part of the cohort changes at each wave, the cohort does not change entirely, as many participants return after missing one or more waves, maintaining similar characteristics to the national cohort. Of note, we did not recruit completely new participants from Rakuten Insight panelists in Wave 8 because the target sample size was fulfilled by recruiting only from participants in past waves. Comparisons between replacement participants (those who did not respond to the previous wave but did participate in the current wave) and dropouts (those who responded to the previous wave but not the current wave) show that, while replacement participants tend to be slightly younger, their demographic and clinical profiles are otherwise similar (Supplementary Table S2A–G).

Data-quality-assurance methods can be broadly divided into two categories: those implemented by Rakuten Insight as part of their standard panel management and those specifically designed by us for this study. Rakuten Insight employs comprehensive quality-control procedures at multiple stages: during recruitment through various channels, at registration with automated fraud-prevention algorithms, and in ongoing participation via introductory surveys, regular qualitative checks, and demographic verification. Additional details on Rakuten Insight’s panel-quality-assurance processes are available in their published documentation [3]. We implemented additional study-specific quality measures by identifying and excluding “satisficers” through the algorithmic detection of inconsistent or unreasonable response patterns and attention checks throughout the survey. The algorithm to detect satisficers was modified for each wave based on the specific items and available data (such as response time) in that wave. Details of the algorithms used in each wave are presented in Table 3.

**Table 3:** Algorithms to identify satisficers in each survey.

Wave	Wrong response in attention check^a^	Using all substances^b^	Having all diseases^c^	More than 15 household members	Response time of <15 minutes
Wave 1	✓	✓	✓		
Wave 2	✓	✓	✓		
Wave 3	✓	✓	✓		
Wave 4	✓	✓	✓		
Wave 5	✓	✓	✓	✓	
Wave 6	✓	✓	✓	✓	
Wave 7	✓	✓	✓	✓	✓
Wave 8	✓	✓	✓	✓	✓

a The question for the attention check was: “Select the second option from the bottom.”

b Participants were asked: For those who selected “Use almost every day” or “Sometimes” for the question “Are you currently consuming or using alcohol or drugs?” please respond for each of the following items: alcohol (such as beer, sake, shochu, wine, or whiskey), sleeping pills or anti-anxiety medications, narcotics such as morphine (prescribed by a doctor for cancer pain), narcotics such as morphine (prescribed by a doctor for pain other than cancer), narcotics such as morphine (obtained by methods not prescribed by a doctor), inhalation of organic solvents such as thinner or toluene (excluding appropriate use for work purposes), dangerous drugs (such as legal highs or magic mushrooms), cannabis (marijuana), and stimulants, cocaine, or heroin.

c Participants were asked: For those who responded “Yes” to all the following items of the question “Do you currently have any chronic illnesses?” please answer each item: 1. Hypertension, 2. Diabetes, 3. Asthma, 4. Bronchitis or Pneumonia, 5. Atopic Dermatitis, 6. Periodontal Disease, 7. Dental Cavities, 8. Otitis Media, 9. Angina, 10. Myocardial Infarction, 11. Stroke (Cerebral Infarction or Cerebral Hemorrhage), 12. COPD (Chronic Obstructive Pulmonary Disease), 13. Cancer or Malignant Tumors, 14. Chronic Pain (such as back pain or headaches lasting >3 months), 15. Depression, 16. Other Mental Disorders besides Depression.

The JACSIS dataset comprises a comprehensive collection of self-reported data gathered through online questionnaires administered twice a year (Table 4). Survey items were determined through multiple iterative discussions among the research team, with careful selection of the most appropriate scales for each construct of interest, balancing participant burden with measurement validity and reliability. Survey items include: demographic information (age, sex, gender identity, sexual orientation, residence); socioeconomic indicators (education, income, marital status, employment status); anthropometric measurements (self-reported height and weight); health-related factors (physical symptoms, medical conditions, treatment status of chronic diseases such as hypertension and diabetes, hospitalization experiences, healthcare utilization); validated psychometric scales [Kessler-6 for depression screening [4], EQ5D-5L for health-related quality of life [5], COPD-Q for respiratory symptoms [6]; lifestyle behaviors (tobacco use with Fagerstrom Test [7], alcohol use with AUDIT [8] and CAGE questionnaires [9], gambling behaviors); COVID-19-specific measures (infection history, COVID-19-like symptoms, preventive behaviors, vaccination status, vaccine attitudes using the Fear of COVID-19 Scale) [10]; and social measures (Lubben Social Network Scale [11], UCLA loneliness scale version3 [12], adverse and positive childhood experiences [13, 14])]. Additionally, parallel information about partners, spouses, and children was collected for relevant items, enabling a thorough analysis of family dynamics and their influence on health and well-being. The dataset includes both cross-sectional and longitudinal data components, with some variables collected at every wave and others at specific intervals based on the research priorities. All measures are obtained via self-report rather than clinical assessments or biological sampling. The first survey wave included ∼600 questions; however, due to branching logic in the questionnaire design, each respondent typically answered ∼100 questions rather than the entire set. In Wave 7, in which the completion times were first measured systematically, the mean survey completion time was 49.4 minutes (standard deviation = 28.5 minutes).

**Table 4:** List of collected variables that were utilized in published studies.

Variables	Wave 1	Wave 2	Wave 3	Wave 4	Wave 5	Wave 6	Wave 7	Wave 8
Demographic factors								
Age	✓	✓	✓	✓	✓	✓	✓	✓
Biological sex assigned at birth	✓	✓	✓	✓	✓	✓	✓	✓
Gender identity					✓			
Sexual orientation					✓		✓	
Weight and height	✓	✓		✓	✓	✓	✓	✓
Residence	✓	✓	✓	✓	✓	✓	✓	✓
Socioeconomic status								
Academic attainment	✓	✓	✓	✓	✓	✓	✓	✓
Marital status	✓	✓	✓	✓	✓	✓	✓	✓
Equivalent household income	✓	✓	✓	✓	✓	✓	✓	✓
Living arrangements	✓	✓	✓	✓	✓	✓	✓	✓
Loan or debt	✓		✓		✓			
Social capital				✓		✓	✓	
Lubben Social Network Scale					✓		✓	✓
Adverse childhood experience			✓	✓	✓		✓	✓
Positive childhood experience					✓		✓	
Financial difficulties				✓		✓		✓
Household factors								
Number of household members	✓	✓	✓	✓	✓	✓	✓	✓
Number of family members	✓	✓	✓	✓	✓	✓	✓	✓
Caregiving (e.g. time, severity)	✓						✓	
Occupational factors								
Current employment situation	✓		✓		✓		✓	
Changes in work arrangements	✓	✓		✓		✓		✓
Industrial sector		✓	✓		✓			
Working at a COVID-19 healthcare worksite		✓						
Time for VDT work	✓	✓	✓					
Work functioning impairment	✓	✓	✓	✓	✓	✓	✓	✓
Health-related factors								
Physical symptoms		✓	✓		✓			
Respiratory symptoms (COPD-Q)						✓		
HPV vaccination attitudes and experiences			✓		✓		✓	
Loneliness				✓	✓		✓	✓
Depression (Kessler-6)	✓	✓	✓	✓	✓	✓	✓	✓
Health-related quality of life (EQ5D-5L)	✓	✓	✓	✓				
Self-rated happiness	✓	✓		✓		✓		✓
Medical conditions	✓	✓	✓	✓	✓	✓	✓	✓
Oral health impact profile					✓			
Somatic symptom scale			✓		✓	✓	✓	✓
Burnout		✓						
Rubella vaccination history					✓		✓	
Vaccination for seasonal influenza	✓	✓		✓			✓	
Usual and problematic behavior								
Alcohol use	✓	✓	✓	✓	✓	✓	✓	✓
Substance use	✓	✓			✓		✓	✓
CAGE questionnaire	✓			✓		✓		✓
Alcohol-use disorders identification test (AUDIT)				✓		✓		
Household expenditures on daily activities	✓	✓		✓	✓			
Preventive behaviors for infection	✓	✓	✓	✓	✓	✓	✓	✓
Child abuse (adversity)	✓							
South Oaks gambling screen						✓		✓
Problem gambling severity index						✓		✓
Tobacco-related factors								
Tobacco product use status	✓	✓	✓	✓	✓	✓	✓	✓
Smoke-free rules at workplace, home, and in car	✓	✓	✓	✓		✓		✓
Trial of smoking cessation		✓		✓		✓		✓
Smoking-cessation stage	✓	✓		✓		✓		✓
Experience of exposure to secondhand smoking	✓			✓		✓		✓
Health beliefs related to tobacco use		✓				✓		✓
Fagerstrom test for nicotine dependence		✓		✓		✓		✓
COVID-19-related factors								
Delayed medical care due to COVID-19 pandemic	✓	✓	✓	✓				
Change in lifestyle after the COVID-19 pandemic	✓							
Fear of COVID-19 (FCV-19S)	✓	✓	✓	✓	✓	✓	✓	
Information source of COVID-19			✓		✓			
Stigmatized beliefs related to COVID-19		✓						
Use of government subsidies for travel (Go To Travel)	✓	✓						
COVID-19 vaccine hesitancy		✓			✓			
Perception of COVID-19 vaccine			✓	✓	✓			
Experiencing COVID-19 infection	✓	✓	✓	✓	✓	✓	✓	
Behaviors during state of emergency	✓							
Use of COVID-19 contact-tracing application	✓	✓	✓					
Behavior change after COVID-19 vaccine			✓					
Others								
Political affiliation				✓				
Trust in government	✓	✓	✓					
Use of mass media as a source of information	✓	✓		✓		✓		✓
Emotional control		✓						
Food-insecurity experience scale			✓					
Vaccination-readiness scale							✓	
Use of children’s cafeteria							✓	

COPD-Q, chronic obstructive pulmonary disease knowledge questionnaire; EQ5D-5L, EuroQol 5 dimensions 5-level; HPV, human papilloma virus; JACSIS, the Japan COVID-19 and Society Internet Survey; VDT, visual display terminal.

Note: All variables were assessed by using self-administered, web-based questionnaires conducted via Rakuten Insight’s secure web-based platform. Variables without a specific scale name were measured by using original items developed by the JACSIS study team. Detailed survey questionnaires are available in Japanese upon request.

The JACSIS dataset is linked with the pre-pandemic JASTIS studies conducted between 2015 and 2020. This linkage enables pre- and post-pandemic comparisons, with 6494 participants from JASTIS 2019 (pre-pandemic) and 7351 from JASTIS 2020 (pandemic onset) also participating in JACSIS 2020. Each research participant is assigned a unique numerical identifier (ID) that is independent of their personal information, with these IDs assigned consecutively from JASTIS 2015 and new IDs generated only for new participants who have never previously participated in any wave of JACSIS or JASTIS. When data are received from Rakuten Insight, survey responses are linked to these IDs, which are then used to merge data across different waves for longitudinal analyses. While these IDs are linked to Rakuten Insight login credentials, the correspondence table between IDs and login information is managed exclusively by Rakuten Insight, with researchers having no access to this identifying information.

## Data resource use

JACSIS has revealed significant socioeconomic disparities in pandemic impacts, healthcare utilization, and adherence to preventive measures, while also highlighting the role of factors such as trust in government and information sources in shaping public responses to the pandemic. The research has provided valuable insights into the effects of changing work environments, the prevalence of mental health issues, and the complexities surrounding COVID-19 vaccine uptake and hesitancy in Japan, with these findings disseminated through 117 published papers by 30 November 2024. The full list of papers is available on the JACSIS website (https://jacsis-study.jp/output/index.html). Our main findings include the following:

- We examined the association between participation in Japan’s subsidy program for domestic travel (“Go To Travel” campaign) and the incidence of COVID-19-like symptoms by using Wave 1 data [15]. We found that participants in the subsidy program (12.9% of respondents) had higher odds of experiencing COVID-19-like symptoms compared with non-participants. For example, participants had 1.83 [95% confidence interval (CI), 1.34–2.48] times higher odds of experiencing high fever and 1.98 (95% CI, 1.15–3.40) times higher odds of smell and taste disorder. Following this study, the Japanese government decided to suspend the Go To Travel campaign in December 2020.
- We investigated the association between worsened socioeconomic conditions due to the COVID-19 pandemic and dental pain in Japan by using Wave 1 data [16]. We found that 9.8% of the participants reported dental pain and those who experienced household income reduction, work reduction, and job loss were more likely to report dental pain [adjusted odds ratios (aOR): 1.42 (95% CI, 1.28–1.57), 1.58 (95% CI, 1.41–1.76), and 2.17 (95% CI, 1.64–2.88), respectively].
- The JACSIS dataset has accumulated a diverse array of variables beyond COVID-19-specific measures, enabling researchers to conduct investigations on a wide spectrum of health behaviors and outcomes unrelated to the pandemic. Current ongoing data analyses from the JACSIS dataset include investigations into the causal relationships between psychotic symptoms and unhealthy behaviors such as smoking, alcohol consumption, and substance use (including the possibility of self-medication behaviors), as well as studies examining procrastination tendencies and the associations between dietary supplement use, health literacy, and medication adherence. Future analyses under consideration include investigations into the economic burden of third-hand smoke exposure and asbestos-related diseases. Additionally, the research team is planning to incorporate new survey items into upcoming waves, including screening tools for prodromal symptoms of schizophrenia, sexual behavior assessments, and stigma scales (the reported and intended behavior scale).

## Strengths and weaknesses

This study boasts several significant strengths. Firstly, it features a large sample size of ∼30 000 participants for each survey wave, providing substantial statistical power and allowing detailed subgroup analyses. The longitudinal follow-up design enables the examination of trends and changes over time, enhancing our understanding of causal relationships. Another major strength is the extensive array of variables collected, offering a comprehensive view of participants’ health, behaviors, and socioeconomic circumstances. Furthermore, the study benefits from the collaboration of researchers from multiple institutions across Japan, bringing together diverse expertise from various disciplines including epidemiology, public health, clinical medicine, statistics, and social sciences.

A unique advantage of this study is its ability to compare data from before and after the COVID-19 pandemic, as a subset of participants had already taken part in JASTIS studies conducted between 2015 and 2020 (January to March). This feature allows valuable insights into the impact of the pandemic on various aspects of participants’ lives. Additionally, the inclusion of gender-identity and sexual-orientation information, collected via online survey—a method validated by a scoping review for these sensitive topics [17]—adds significant value to the dataset, enabling research on often understudied populations.

Despite its strengths, this study has several weaknesses. The primary weakness is the potential for sampling bias inherent in internet-based surveys. As we lack information about individuals who are not members of the Rakuten Insight panel, our sample may not be fully representative of the general population. However, we can attempt to mitigate this issue through the application of weighting techniques. The details of weighting techniques are provided in the Supplementary Method.

Another weakness is that our data cannot provide medically verified objective measures due to the self-reported nature of online surveys. While our questionnaires attempt to capture both subjective experiences (such as fear of COVID-19, perceived risk, and mental health impacts) and objective indicators (such as fever, employment status changes, and receipt of government subsidies), the validity of objective measures is inherently limited by the absence of linkage with healthcare-facility data or public administrative databases. For research questions requiring clinical validation or verified administrative data, investigators should utilize appropriate medical or governmental data sources rather than self-reported survey responses. However, this limitation does not diminish the value of our dataset, especially for capturing population-level changes in subjective experiences, perceptions, and behaviors during the COVID-19 pandemic.

Third, the timing of the baseline data collection presents potential reverse-causality concerns. As JACSIS recruitment began in August 2020, 6 months after the COVID-19 pandemic started in Japan, the baseline characteristics may already reflect pandemic-induced changes. However, our study addresses this through questionnaire items tracking life changes (divorce, job changes, educational disruptions) and data linkage with pre-pandemic JASTIS studies.

Lastly, as with all self-reported data, there is a possibility of recall bias or social-desirability bias in participants’ responses, which could affect the accuracy of some subjective measures.

## Data resource access

Access to the data is governed by the study investigators and requires permission. Researchers interested in using the data should submit a research proposal that is consistent with ethical approval, confidentiality agreements, and data-management protocols.

To apply for data access or to obtain more information about the study, please contact the principal investigator Dr Takahiro Tabuchi (tabuchitak@gmail.com). Dr Tabuchi can provide further details about the application process, available data, and potential collaborations.

## Ethics Approval

The JACSIS study received initial approval from the Institutional Review Board of the Osaka International Cancer Institute on 19 June 2020 (approval number: 20084). Following the principal investigator’s institutional move, the study also obtained approval from the Ethics Committee of Tohoku University Graduate School of Medicine [approval numbers: 2024-1-231 (27 June 2024) and 2024-1-517 (22 October 2024)]. The JASTIS study was approved by the Research Ethics Committee of the Osaka International Cancer Institute (approval number: 1412175183). For both studies, all participants provided informed consent before taking part in the survey. The consent process was conducted online and participants were required to agree to the terms of the study before they could proceed with answering the survey questions. For obtaining the consent of participants aged 16–17 years, see details in the “Data resource basics” section. The writing of this cohort profile was approved by the Kyoto University Graduate School and Faculty of Medicine Ethics Committee [approval number: R4810 (9 December 2024)].

## Supplementary Material

dyag025_Supplementary_Data

## Data Availability

The data underlying this article will be shared on reasonable request to the corresponding author, subject to approval by the study investigators and in accordance with ethical approval, confidentiality agreements, and data-management protocols. Researchers wishing to access the data should submit a research proposal. Requests and enquiries should be addressed to the principal investigator, Dr Takahiro Tabuchi (tabuchitak@gmail.com).
