# Supplementary material for: Data Resource Profile: The Japan COVID-19 and Society Internet Survey (JACSIS)
Source: Int J Epidemiol. 2026 Mar 10;55(2):dyag025. doi: 10.1093/ije/dyag025 (PMC13017115; doi:10.1093/ije/dyag025)
Supplement: dyag025_Supplementary_Data [file dyag025_supplementary_data.docx]

Table of contents

[Supplementary Table S1 Characteristics of the cohort from waves 2 to 7 compared with the Japanese general population 1](#_Toc215227351)

[(a) Wave 2 1](#_Toc215227352)

[(b) Wave 3 3](#_Toc215227353)

[(c) Wave 4 6](#_Toc215227354)

[(d) Wave 5 8](#_Toc215227355)

[(e) Wave 6 11](#_Toc215227356)

[(f) Wave 7 13](#_Toc215227357)

[Supplementary Table S2 Comparison of characteristics between newly added and retained participants at waves 2–8 15](#_Toc215227358)

[(a) Wave 2 16](#_Toc215227359)

[(b) Wave 3 18](#_Toc215227360)

[(c) Wave 4 20](#_Toc215227361)

[(d) Wave 5 23](#_Toc215227362)

[(e) Wave 6 25](#_Toc215227363)

[(f) Wave 7 28](#_Toc215227364)

[(g) Wave 8 30](#_Toc215227365)

[Supplementary Table S3 Algorithms to identify satisficers in each survey wave 32](#_Toc215227366)

[Supplementary Method 33](#_Toc215227367)

# Supplementary Table S1 Characteristics of the cohort from waves 2 to 7 compared with the Japanese general population

## Wave 2

|  | **Wave2 (Unweighted)** | | **Wave2 (Weighted)** | | **%Japanese population** |
| --- | --- | --- | --- | --- | --- |
| **Characteristic** | **N = 23,142**^1^ | | **N = 23,142**^1^ | |  |
| Age (median, IQR) | 51 (37, 65) | | 51 (38, 65) | | 46.7 |
| Age group |  | |  | |  |
| 0–15 | 0 (0%) | | 0 (0%) | | 12.1 |
| 16–19 | 611 (2.6%) | | 611 (2.6%) | | 4.5 |
| 20–29 | 2,781 (12%) | | 2,781 (12%) | | 10.4 |
| 30–39 | 3,181 (14%) | | 3,181 (14%) | | 11.6 |
| 40–49 | 4,502 (19%) | | 4,502 (19%) | | 14.8 |
| 50–59 | 4,211 (18%) | | 4,211 (18%) | | 13.2 |
| 60–69 | 4,257 (18%) | | 4,257 (18%) | | 11.1 |
| 70–79 | 3,518 (15%) | | 3,538 (15%) | | 11.7 |
| 80– | 81 (0.4%) | | 61 (0.3%) | | 7.8 |
| Sex |  | |  | |  |
| Female | 11,376 (49%) | | 11,376 (49%) | | 48.7 |
| Male | 11,766 (51%) | | 11,766 (51%) | | 51.3 |
| Living area |  | |  | |  |
| Hokkaido and Tohoku | 2,554 (11%) | | 3,285 (14%) | | 9.2 |
| Kanto | 8,226 (36%) | | 4,503 (19%) | | 41 |
| Hokuriku and Ko-shin-etsu | 1,399 (6.0%) | | 2,539 (11%) | | 4 |
| Tokai | 2,735 (12%) | | 2,707 (12%) | | 11.1 |
| Kansai | 3,894 (17%) | | 3,344 (14%) | | 19 |
| Chugoku and Shikoku | 1,973 (8.5%) | | 3,837 (17%) | | 6.1 |
| Kyushu and Okinawa | 2,361 (10%) | | 2,928 (13%) | | 9.4 |
| Academic attainment |  | |  | |  |
| Junior high school graduate | 793 (3.4%) | | 1,782 (7.7%) | | 13.1 |
| High school graduate | 7,312 (32%) | | 11,546 (50%) | | 44.2 |
| College graduate | 4,762 (21%) | | 3,669 (16%) | | 16.2 |
| Bachelor's degree | 9,230 (40%) | | 4,231 (18%) | | 23.1 |
| Master's or doctoral degree | 1,003 (4.3%) | | 1,838 (7.9%) | | 2.4 |
| Other | 42 (0.2%) | | 77 (0.3%) | | 0 |
| Marital status |  | |  | |  |
| Married | 14,045 (61%) | | 15,262 (66%) | | 58.3 |
| Never married | 6,859 (30%) | | 5,697 (25%) | | 27.4 |
| Widowed or divorced | 2,238 (9.7%) | | 2,183 (9.4%) | | 14.2 |
| Number of households |  | |  | |  |
| 1 | 4,504 (19%) | | 3,535 (15%) | | 38 |
| 2 | 8,065 (35%) | | 8,121 (35%) | | 28.1 |
| 3 | 5,248 (23%) | | 5,274 (23%) | | 16.6 |
| 4 | 3,719 (16%) | | 4,079 (18%) | | 12.0 |
| 5+ | 1,606 (6.9%) | | 2,132 (9.2%) | | 5.5 |
| Household equivalent income |  | |  | |  |
| Low | 1,987 (8.6%) | | 2,019 (8.7%) | | 19 |
| Medium | 4,845 (21%) | | 5,094 (22%) | | 26.4 |
| High | 11,622 (50%) | | 10,812 (47%) | | 54.6 |
| Unknown/Declined to answer | 4,688 (20%) | | 5,217 (23%) | | 0 |
| Type of employment |  | |  | |  |
| Employer | 735 (3.2%) | | 681 (2.9%) | | 2.8 |
| Self-employed | 1,340 (5.8%) | | 1,391 (6.0%) | | 6.1 |
| Regular employee | 7,955 (34%) | | 7,451 (32%) | | 30.1 |
| Non regular employee | 4,285 (19%) | | 4,613 (20%) | | 19.1 |
| Unemployed | 8,827 (38%) | | 9,006 (39%) | | 41.1 |
| ^1^Median (Q1, Q3); n (%) | |  | |  |  |

## Wave 3

|  | **Wave3 (Unweighted)** | | **Wave3 (Weighted)** | **%Japanese population** |
| --- | --- | --- | --- | --- |
| **Characteristic** | **N = 28,175**^1^ | | **N = 28,175**^1^ |  |
| Age (median, IQR) | 50 (37, 65) | | 50 (37, 65) | 46.7 |
| Age group |  | |  |  |
| 0–15 | 0 (0%) | | 0 (0%) | 12.1 |
| 16–19 | 573 (2.0%) | | 573 (2.0%) | 4.5 |
| 20–29 | 3,626 (13%) | | 3,626 (13%) | 10.4 |
| 30–39 | 4,149 (15%) | | 4,149 (15%) | 11.6 |
| 40–49 | 5,454 (19%) | | 5,454 (19%) | 14.8 |
| 50–59 | 4,785 (17%) | | 4,785 (17%) | 13.2 |
| 60–69 | 4,882 (17%) | | 4,882 (17%) | 11.1 |
| 70–79 | 4,527 (16%) | | 4,529 (16%) | 11.7 |
| 80– | 179 (0.6%) | | 177 (0.6%) | 7.8 |
| Sex |  | |  |  |
| Female | 14,305 (51%) | | 14,305 (51%) | 48.7 |
| Male | 13,870 (49%) | | 13,870 (49%) | 51.3 |
| Living area |  | |  |  |
| Hokkaido and Tohoku | 3,098 (11%) | | 3,750 (13%) | 9.2 |
| Kanto | 9,948 (35%) | | 6,864 (24%) | 41 |
| Hokuriku and Ko-shin-etsu | 1,656 (5.9%) | | 2,474 (8.8%) | 4 |
| Tokai | 3,427 (12%) | | 3,632 (13%) | 11.1 |
| Kansai | 4,608 (16%) | | 4,031 (14%) | 19 |
| Chugoku and Shikoku | 2,407 (8.5%) | | 3,697 (13%) | 6.1 |
| Kyushu and Okinawa | 3,031 (11%) | | 3,727 (13%) | 9.4 |
| Academic attainment |  | |  |  |
| Junior high school graduate | 757 (2.7%) | | 1,719 (6.1%) | 13.1 |
| High school graduate | 8,890 (32%) | | 14,716 (52%) | 44.2 |
| College graduate | 5,903 (21%) | | 5,245 (19%) | 16.2 |
| Bachelor's degree | 11,206 (40%) | | 5,676 (20%) | 23.1 |
| Master's or doctoral degree | 1,253 (4.4%) | | 533 (1.9%) | 2.4 |
| Other | 166 (0.6%) | | 287 (1.0%) | 0 |
| Marital status |  | |  |  |
| Married | 16,969 (60%) | | 17,801 (63%) | 58.3 |
| Never married | 8,322 (30%) | | 7,122 (25%) | 27.4 |
| Widowed or divorced | 2,884 (10%) | | 3,252 (12%) | 14.2 |
| Number of households |  | |  |  |
| 1 | 5,910 (21%) | | 4,618 (16%) | 38 |
| 2 | 9,699 (34%) | | 9,372 (33%) | 28.1 |
| 3 | 6,413 (23%) | | 6,674 (24%) | 16.6 |
| 4 | 4,348 (15%) | | 5,035 (18%) | 12.0 |
| 5+ | 1,805 (6.4%) | | 2,476 (8.8%) | 5.5 |
| Household equivalent income |  | |  |  |
| Low | 2,415 (8.6%) | | 2,698 (9.6%) | 19 |
| Medium | 5,738 (20%) | | 5,618 (20%) | 26.4 |
| High | 13,974 (50%) | | 13,130 (47%) | 54.6 |
| Unknown/Declined to answer | 6,048 (21%) | | 6,729 (24%) | 0 |
| Type of employment |  | |  |  |
| Employer | 990 (3.5%) | | 912 (3.2%) | 2.8 |
| Self-employed | 1,206 (4.3%) | | 1,221 (4.3%) | 6.1 |
| Regular employee | 9,722 (35%) | | 9,210 (33%) | 30.1 |
| Non regular employee | 6,399 (23%) | | 6,514 (23%) | 19.1 |
| Unemployed | 9,858 (35%) | | 10,318 (37%) | 41.1 |
| ^1^Median (Q1, Q3); n (%) | |  |  |  |

## Wave 4

|  | **Wave4 (Unweighted)** | | **Wave4 (Weighted)** | **%Japanese population** |
| --- | --- | --- | --- | --- |
| **Characteristic** | **N = 28,175**^1^ | | **N = 28,175**^1^ |  |
| Age (median, IQR) | 50 (37, 65) | | 50 (37, 65) | 46.7 |
| Age group |  | |  |  |
| 0-15 | 0 (0%) | | 0 (0%) | 12.1 |
| 16–19 | 573 (2.0%) | | 573 (2.0%) | 4.5 |
| 20–29 | 3,626 (13%) | | 3,626 (13%) | 10.4 |
| 30–39 | 4,149 (15%) | | 4,149 (15%) | 11.6 |
| 40–49 | 5,454 (19%) | | 5,454 (19%) | 14.8 |
| 50–59 | 4,785 (17%) | | 4,785 (17%) | 13.2 |
| 60–69 | 4,882 (17%) | | 4,882 (17%) | 11.1 |
| 70–79 | 4,527 (16%) | | 4,529 (16%) | 11.7 |
| 80– | 179 (0.6%) | | 177 (0.6%) | 7.8 |
| Sex |  | |  |  |
| Female | 14,305 (51%) | | 14,305 (51%) | 48.7 |
| Male | 13,870 (49%) | | 13,870 (49%) | 51.3 |
| Living area |  | |  |  |
| Hokkaido and Tohoku | 3,098 (11%) | | 3,750 (13%) | 9.2 |
| Kanto | 9,948 (35%) | | 6,864 (24%) | 41 |
| Hokuriku and Ko-shin-etsu | 1,656 (5.9%) | | 2,474 (8.8%) | 4 |
| Tokai | 3,427 (12%) | | 3,632 (13%) | 11.1 |
| Kansai | 4,608 (16%) | | 4,031 (14%) | 19 |
| Chugoku and Shikoku | 2,407 (8.5%) | | 3,697 (13%) | 6.1 |
| Kyushu and Okinawa | 3,031 (11%) | | 3,727 (13%) | 9.4 |
| Academic attainment |  | |  |  |
| Junior high school graduate | 757 (2.7%) | | 1,719 (6.1%) | 13.1 |
| High school graduate | 8,890 (32%) | | 14,716 (52%) | 44.2 |
| College graduate | 5,903 (21%) | | 5,245 (19%) | 16.2 |
| Bachelor's degree | 11,206 (40%) | | 5,676 (20%) | 23.1 |
| Master's or doctoral degree | 1,253 (4.4%) | | 533 (1.9%) | 2.4 |
| Other | 166 (0.6%) | | 287 (1.0%) | 0 |
| Marital status |  | |  |  |
| Married | 16,969 (60%) | | 17,801 (63%) | 58.3 |
| Never married | 8,322 (30%) | | 7,122 (25%) | 27.4 |
| Widowed or divorced | 2,884 (10%) | | 3,252 (12%) | 14.2 |
| Number of households |  | |  |  |
| 1 | 5,910 (21%) | | 4,618 (16%) | 38 |
| 2 | 9,699 (34%) | | 9,372 (33%) | 28.1 |
| 3 | 6,413 (23%) | | 6,674 (24%) | 16.6 |
| 4 | 4,348 (15%) | | 5,035 (18%) | 12.0 |
| 5+ | 1,805 (6.4%) | | 2,476 (8.8%) | 5.5 |
| Household equivalent income |  | |  |  |
| Low | 2,415 (8.6%) | | 2,698 (9.6%) | 19 |
| Medium | 5,738 (20%) | | 5,618 (20%) | 26.4 |
| High | 13,974 (50%) | | 13,130 (47%) | 54.6 |
| Unknown/Declined to answer | 6,048 (21%) | | 6,729 (24%) | 0 |
| Type of employment |  | |  |  |
| Employer | 990 (3.5%) | | 912 (3.2%) | 2.8 |
| Self-employed | 1,206 (4.3%) | | 1,221 (4.3%) | 6.1 |
| Regular employee | 9,722 (35%) | | 9,210 (33%) | 30.1 |
| Non regular employee | 6,399 (23%) | | 6,514 (23%) | 19.1 |
| Unemployed | 9,858 (35%) | | 10,318 (37%) | 41.1 |
| ^1^Median (Q1, Q3); n (%) | |  |  |  |

## Wave 5

|  | **Wave5 (Unweighted)** | | **Wave5 (Weighted)** | **%Japanese population** |
| --- | --- | --- | --- | --- |
| **Characteristic** | **N = 28,630**^1^ | | **N = 28,630**^1^ |  |
| Age (median, IQR) | 46 (34, 63) | | 46 (34, 63) | 46.7 |
| Age group |  | |  |  |
| 0–15 | 0 (0%) | | 0 (0%) | 12.1 |
| 16–19 | 225 (0.8%) | | 225 (0.8%) | 4.5 |
| 20–29 | 4,575 (16%) | | 4,575 (16%) | 10.4 |
| 30–39 | 6,356 (22%) | | 6,356 (22%) | 11.6 |
| 40–49 | 4,781 (17%) | | 4,781 (17%) | 14.8 |
| 50–59 | 4,145 (14%) | | 4,145 (14%) | 13.2 |
| 60–69 | 4,245 (15%) | | 4,245 (15%) | 11.1 |
| 70–79 | 3,940 (14%) | | 3,895 (14%) | 11.7 |
| 80– | 363 (1.3%) | | 408 (1.4%) | 7.8 |
| Sex |  | |  |  |
| Female | 14,628 (51%) | | 14,628 (51%) | 48.7 |
| Male | 14,002 (49%) | | 14,002 (49%) | 51.3 |
| Living area |  | |  |  |
| Hokkaido and Tohoku | 3,038 (11%) | | 3,671 (13%) | 9.2 |
| Kanto | 10,254 (36%) | | 7,201 (25%) | 41 |
| Hokuriku and Ko-shin-etsu | 1,595 (5.6%) | | 2,412 (8.4%) | 4 |
| Tokai | 3,522 (12%) | | 3,897 (14%) | 11.1 |
| Kansai | 4,944 (17%) | | 4,186 (15%) | 19 |
| Chugoku and Shikoku | 2,432 (8.5%) | | 3,667 (13%) | 6.1 |
| Kyushu and Okinawa | 2,845 (9.9%) | | 3,596 (13%) | 9.4 |
| Academic attainment |  | |  |  |
| Junior high school graduate | 496 (1.7%) | | 1,274 (4.4%) | 13.1 |
| High school graduate | 7,272 (25%) | | 14,182 (50%) | 44.2 |
| College graduate | 6,131 (21%) | | 5,504 (19%) | 16.2 |
| Bachelor's degree | 13,009 (45%) | | 6,608 (23%) | 23.1 |
| Master's or doctoral degree | 1,511 (5.3%) | | 649 (2.3%) | 2.4 |
| Other | 211 (0.7%) | | 413 (1.4%) | 0 |
| Marital status |  | |  |  |
| Married | 17,793 (62%) | | 17,810 (62%) | 58.3 |
| Never married | 8,346 (29%) | | 7,885 (28%) | 27.4 |
| Widowed or divorced | 2,491 (8.7%) | | 2,935 (10%) | 14.2 |
| Number of households |  | |  |  |
| 1 | 5,817 (20%) | | 4,605 (16%) | 38 |
| 2 | 8,945 (31%) | | 8,904 (31%) | 28.1 |
| 3 | 7,014 (24%) | | 7,157 (25%) | 16.6 |
| 4 | 4,885 (17%) | | 5,284 (18%) | 12.0 |
| 5+ | 1,969 (6.9%) | | 2,680 (9.4%) | 5.5 |
| Household equivalent income |  | |  |  |
| Low | 2,349 (8.2%) | | 2,634 (9.2%) | 19 |
| Medium | 5,612 (20%) | | 5,776 (20%) | 26.4 |
| High | 15,048 (53%) | | 13,566 (47%) | 54.6 |
| Unknown/Declined to answer | 5,621 (20%) | | 6,654 (23%) | 0 |
| Type of employment |  | |  |  |
| Employer | 1,078 (3.8%) | | 1,001 (3.5%) | 2.8 |
| Self-employed | 1,126 (3.9%) | | 1,207 (4.2%) | 6.1 |
| Regular employee | 10,965 (38%) | | 10,307 (36%) | 30.1 |
| Non regular employee | 6,243 (22%) | | 6,634 (23%) | 19.1 |
| Unemployed | 9,218 (32%) | | 9,482 (33%) | 41.1 |
| ^1^Median (Q1, Q3); n (%) | |  |  |  |

## Wave 6

|  | **UnweightedWave6** | | **WeightedWave6** | **%Japanese population** |
| --- | --- | --- | --- | --- |
| **Characteristic** | **N = 31,037**^1^ | | **N = 31,037**^1^ |  |
| Age (median, IQR) | 46 (33, 62) | | 46 (33, 62) | 46.7 |
| Age group |  | |  |  |
| 0–15 | 0 (0%) | | 0 (0%) | 12.1 |
| 16–19 | 722 (2.3%) | | 722 (2.3%) | 4.5 |
| 20–29 | 5,487 (18%) | | 5,487 (18%) | 10.4 |
| 30–39 | 5,752 (19%) | | 5,752 (19%) | 11.6 |
| 40–49 | 5,485 (18%) | | 5,485 (18%) | 14.8 |
| 50–59 | 4,573 (15%) | | 4,573 (15%) | 13.2 |
| 60–69 | 4,635 (15%) | | 4,635 (15%) | 11.1 |
| 70–79 | 3,931 (13%) | | 3,831 (12%) | 11.7 |
| 80– | 452 (1.5%) | | 552 (1.8%) | 7.8 |
| Sex |  | |  |  |
| Female | 15,694 (51%) | | 15,694 (51%) | 48.7 |
| Male | 15,343 (49%) | | 15,343 (49%) | 51.3 |
| Living area |  | |  |  |
| Hokkaido and Tohoku | 3,285 (11%) | | 4,092 (13%) | 9.2 |
| Kanto | 11,229 (36%) | | 7,871 (25%) | 41 |
| Hokuriku and Ko-shin-etsu | 1,671 (5.4%) | | 2,637 (8.5%) | 4 |
| Tokai | 3,827 (12%) | | 4,068 (13%) | 11.1 |
| Kansai | 5,427 (17%) | | 4,627 (15%) | 19 |
| Chugoku and Shikoku | 2,651 (8.5%) | | 3,884 (13%) | 6.1 |
| Kyushu and Okinawa | 2,947 (9.5%) | | 3,858 (12%) | 9.4 |
| Academic attainment |  | |  |  |
| Junior high school graduate | 652 (2.1%) | | 1,445 (4.7%) | 13.1 |
| High school graduate | 7,981 (26%) | | 15,338 (49%) | 44.2 |
| College graduate | 6,480 (21%) | | 5,854 (19%) | 16.2 |
| Bachelor's degree | 14,049 (45%) | | 7,268 (23%) | 23.1 |
| Master's or doctoral degree | 1,632 (5.3%) | | 685 (2.2%) | 2.4 |
| Other | 243 (0.8%) | | 447 (1.4%) | 0 |
| Marital status |  | |  |  |
| Married | 18,568 (60%) | | 18,889 (61%) | 58.3 |
| Never married | 9,735 (31%) | | 9,056 (29%) | 27.4 |
| Widowed or divorced | 2,734 (8.8%) | | 3,092 (10.0%) | 14.2 |
| Number of households |  | |  |  |
| 1 | 6,991 (23%) | | 5,687 (18%) | 38 |
| 2 | 9,582 (31%) | | 9,320 (30%) | 28.1 |
| 3 | 7,130 (23%) | | 7,358 (24%) | 16.6 |
| 4 | 5,194 (17%) | | 5,801 (19%) | 12.0 |
| 5+ | 2,140 (6.9%) | | 2,871 (9.3%) | 5.5 |
| Household equivalent income |  | |  |  |
| Low | 3,039 (9.8%) | | 3,367 (11%) | 19 |
| Medium | 5,917 (19%) | | 5,861 (19%) | 26.4 |
| High | 15,360 (49%) | | 14,323 (46%) | 54.6 |
| Unknown/Declined to answer | 6,721 (22%) | | 7,485 (24%) | 0 |
| Type of employment |  | |  |  |
| Employer | 965 (3.1%) | | 882 (2.8%) | 2.8 |
| Self-employed | 1,171 (3.8%) | | 1,229 (4.0%) | 6.1 |
| Regular employee | 11,968 (39%) | | 11,351 (37%) | 30.1 |
| Non regular employee | 7,030 (23%) | | 7,317 (24%) | 19.1 |
| Unemployed | 9,903 (32%) | | 10,259 (33%) | 41.1 |
| ^1^Median (Q1, Q3); n (%) | |  |  |  |

## Wave 7

|  | **Wave7 (Unweighted)** | | **Wave7 (Weighted)** | **%Japanese population** |
| --- | --- | --- | --- | --- |
| **Characteristic** | **N = 28,481**^1^ | | **N = 28,481**^1^ |  |
| Age (median, IQR) | 48 (32, 64) | | 48 (32, 64) | 46.7 |
| Age group |  | |  |  |
| 0–15 | 0 (0%) | | 0 (0%) | 12.1 |
| 16–19 | 962 (3.4%) | | 962 (3.4%) | 4.5 |
| 20–29 | 5,299 (19%) | | 5,299 (19%) | 10.4 |
| 30–39 | 4,244 (15%) | | 4,244 (15%) | 11.6 |
| 40–49 | 4,513 (16%) | | 4,513 (16%) | 14.8 |
| 50–59 | 4,376 (15%) | | 4,376 (15%) | 13.2 |
| 60–69 | 4,511 (16%) | | 4,511 (16%) | 11.1 |
| 70–79 | 4,054 (14%) | | 4,003 (14%) | 11.7 |
| 80– | 522 (1.8%) | | 573 (2.0%) | 7.8 |
| Sex |  | |  |  |
| Female | 14,410 (51%) | | 14,410 (51%) | 48.7 |
| Male | 14,071 (49%) | | 14,071 (49%) | 51.3 |
| Living area |  | |  |  |
| Hokkaido and Tohoku | 3,090 (11%) | | 3,408 (12%) | 9.2 |
| Kanto | 10,856 (38%) | | 7,616 (27%) | 41 |
| Hokuriku and Ko-shin-etsu | 1,593 (5.6%) | | 2,459 (8.6%) | 4 |
| Tokai | 3,508 (12%) | | 3,755 (13%) | 11.1 |
| Kansai | 4,718 (17%) | | 4,341 (15%) | 19 |
| Chugoku and Shikoku | 2,224 (7.8%) | | 3,505 (12%) | 6.1 |
| Kyushu and Okinawa | 2,492 (8.7%) | | 3,396 (12%) | 9.4 |
| Academic attainment |  | |  |  |
| Junior high school graduate | 540 (1.9%) | | 1,291 (4.5%) | 13.1 |
| High school graduate | 7,485 (26%) | | 14,387 (51%) | 44.2 |
| College graduate | 5,922 (21%) | | 5,328 (19%) | 16.2 |
| Bachelor's degree | 12,906 (45%) | | 6,427 (23%) | 23.1 |
| Master's or doctoral degree | 1,449 (5.1%) | | 593 (2.1%) | 2.4 |
| Other | 179 (0.6%) | | 454 (1.6%) | 0 |
| Marital status |  | |  |  |
| Married | 16,014 (56%) | | 17,108 (60%) | 58.3 |
| Never married | 9,848 (35%) | | 8,450 (30%) | 27.4 |
| Widowed or divorced | 2,619 (9.2%) | | 2,923 (10%) | 14.2 |
| Number of households |  | |  |  |
| 1 | 6,616 (23%) | | 5,029 (18%) | 38 |
| 2 | 9,614 (34%) | | 9,493 (33%) | 28.1 |
| 3 | 6,273 (22%) | | 6,507 (23%) | 16.6 |
| 4 | 4,289 (15%) | | 4,971 (17%) | 12.0 |
| 5+ | 1,689 (5.9%) | | 2,480 (8.7%) | 5.5 |
| Household equivalent income |  | |  |  |
| Low | 2,459 (8.6%) | | 3,023 (11%) | 19 |
| Medium | 5,619 (20%) | | 5,252 (18%) | 26.4 |
| High | 13,827 (49%) | | 12,089 (42%) | 54.6 |
| Unknown/Declined to answer | 6,576 (23%) | | 8,117 (28%) | 0 |
| Type of employment |  | |  |  |
| Employer | 1,111 (3.9%) | | 1,168 (4.1%) | 2.8 |
| Self-employed | 1,099 (3.9%) | | 1,106 (3.9%) | 6.1 |
| Regular employee | 10,468 (37%) | | 9,523 (33%) | 30.1 |
| Non regular employee | 6,640 (23%) | | 6,810 (24%) | 19.1 |
| Unemployed | 9,163 (32%) | | 9,874 (35%) | 41.1 |
| ^1^Median (Q1, Q3); n (%) | |  |  |  |

# Supplementary Table S2 Comparison of characteristics between newly added and retained participants at waves 2–8

## Wave 2

| **Characteristic** | **Dropouts from Wave 1**  (n=5160)^1^ | **Newly added in Wave 2**  (n=3160)^1^ |
| --- | --- | --- |
| Age (median, IQR) | 39 (26, 56) | 44 (30, 56) |
| Age group |  |  |
| 16–19 | 585 (11%) | 103 (3.3%) |
| 20–29 | 1,031 (20%) | 684 (22%) |
| 30–39 | 977 (19%) | 547 (17%) |
| 40–49 | 838 (16%) | 623 (20%) |
| 50–59 | 627 (12%) | 605 (19%) |
| 60–69 | 493 (9.6%) | 543 (17%) |
| 70–79 | 609 (12%) | 55 (1.7%) |
| Sex |  |  |
| Female | 2,860 (55%) | 1,566 (50%) |
| Male | 2,300 (45%) | 1,594 (50%) |
| Living area |  |  |
| Hokkaido and Tohoku | 575 (11%) | 390 (12%) |
| Kanto | 1,790 (35%) | 1,132 (36%) |
| Hokuriku and Ko-shin-etsu | 341 (6.6%) | 162 (5.1%) |
| Tokai | 627 (12%) | 391 (12%) |
| Kansai | 788 (15%) | 605 (19%) |
| Chugoku and Shikoku | 428 (8.3%) | 255 (8.1%) |
| Kyushu and Okinawa | 611 (12%) | 225 (7.1%) |
| Academic attainment |  |  |
| Junior high school graduate | 337 (6.5%) | 124 (3.9%) |
| High school graduate | 1,849 (36%) | 974 (31%) |
| College graduate | 1,082 (21%) | 630 (20%) |
| Bachelor's degree | 1,682 (33%) | 1,274 (40%) |
| Master's or doctoral degree | 189 (3.7%) | 152 (4.8%) |
| Other | 21 (0.4%) | 6 (0.2%) |
| Marital status |  |  |
| Married | 2,744 (53%) | 1,686 (53%) |
| Never married | 1,978 (38%) | 1,266 (40%) |
| Widowed or divorced | 438 (8.5%) | 208 (6.6%) |
| Number of households |  |  |
| 1 | 1,100 (21%) | 656 (21%) |
| 2 | 1,503 (29%) | 894 (28%) |
| 3 | 1,089 (21%) | 761 (24%) |
| 4 | 969 (19%) | 609 (19%) |
| 5+ | 499 (9.7%) | 240 (7.6%) |
| Household equivalent income |  |  |
| Low | 533 (10%) | 259 (8.2%) |
| Medium | 1,068 (21%) | 584 (18%) |
| High | 2,424 (47%) | 1,615 (51%) |
| Unknown/Declined to answer | 1,135 (22%) | 702 (22%) |
| Type of employment |  |  |
| Employer | 159 (3.1%) | 117 (3.7%) |
| Self-employed | 244 (4.7%) | 180 (5.7%) |
| Regular employee | 1,804 (35%) | 1,303 (41%) |
| Non regular employee | 840 (16%) | 665 (21%) |
| Unemployed | 2,113 (41%) | 895 (28%) |
| ^1^Median (Q1, Q3); n (%) |  |  |

## Wave 3

| **Characteristic** | **Dropouts from Wave 2** (n = 6660)^1^ | **Newly added in Wave 3** (n = 11660)^1^ |
| --- | --- | --- |
| Age (median, IQR) | 44 (30, 58) | 44 (31, 60) |
| Age group |  |  |
| 16–19 | 334 (5.0%) | 424 (3.6%) |
| 20–29 | 1,258 (19%) | 2,069 (18%) |
| 30–39 | 1,202 (18%) | 2,242 (19%) |
| 40–49 | 1,277 (19%) | 2,361 (20%) |
| 50–59 | 1,084 (16%) | 1,587 (14%) |
| 60–69 | 1,003 (15%) | 1,584 (14%) |
| 70–79 | 490 (7.4%) | 1,377 (12%) |
| 80– | 12 (0.2%) | 16 (0.1%) |
| Sex |  |  |
| Female | 3,400 (51%) | 6,264 (54%) |
| Male | 3,260 (49%) | 5,396 (46%) |
| Living area |  |  |
| Hokkaido and Tohoku | 705 (11%) | 1,239 (11%) |
| Kanto | 2,385 (36%) | 4,159 (36%) |
| Hokuriku and Ko-shin-etsu | 401 (6.0%) | 610 (5.2%) |
| Tokai | 790 (12%) | 1,480 (13%) |
| Kansai | 1,148 (17%) | 1,870 (16%) |
| Chugoku and Shikoku | 587 (8.8%) | 1,016 (8.7%) |
| Kyushu and Okinawa | 644 (9.7%) | 1,286 (11%) |
| Academic attainment |  |  |
| Junior high school graduate | 322 (4.8%) | 355 (3.0%) |
| High school graduate | 2,183 (33%) | 3,727 (32%) |
| College graduate | 1,324 (20%) | 2,497 (21%) |
| Bachelor's degree | 2,525 (38%) | 4,499 (39%) |
| Master's or doctoral degree | 292 (4.4%) | 493 (4.2%) |
| Other | 14 (0.2%) | 89 (0.8%) |
| Marital status |  |  |
| Married | 3,724 (56%) | 6,573 (56%) |
| Never married | 2,445 (37%) | 4,020 (34%) |
| Widowed or divorced | 491 (7.4%) | 1,067 (9.2%) |
| Number of households |  |  |
| 1 | 1,349 (20%) | 2,620 (22%) |
| 2 | 2,013 (30%) | 3,671 (31%) |
| 3 | 1,551 (23%) | 2,682 (23%) |
| 4 | 1,200 (18%) | 1,887 (16%) |
| 5+ | 547 (8.2%) | 800 (6.9%) |
| Household equivalent income |  |  |
| Low | 574 (8.6%) | 1,043 (8.9%) |
| Medium | 1,290 (19%) | 2,255 (19%) |
| High | 3,327 (50%) | 5,832 (50%) |
| Unknown/Declined to answer | 1,469 (22%) | 2,530 (22%) |
| Type of employment |  |  |
| Employer | 237 (3.6%) | 403 (3.5%) |
| Self-employed | 349 (5.2%) | 427 (3.7%) |
| Regular employee | 2,545 (38%) | 4,368 (37%) |
| Non regular employee | 1,283 (19%) | 2,856 (24%) |
| Unemployed | 2,246 (34%) | 3,606 (31%) |
| ^1^Median (Q1, Q3); n (%) |  |  |

## Wave 4

| **Characteristic** | **Dropouts in Wave 3** (n = 4134)^1^ | **Newly added in Wave 4**  (n = 6134)^1^ |
| --- | --- | --- |
| Age (median, IQR) | 42 (28, 57) | 26 (22, 37) |
| Age group |  |  |
| 16–19 | 219 (5.3%) | 454 (7.4%) |
| 20–29 | 953 (23%) | 3,663 (60%) |
| 30–39 | 751 (18%) | 652 (11%) |
| 40–49 | 708 (17%) | 506 (8.2%) |
| 50–59 | 562 (14%) | 312 (5.1%) |
| 60–69 | 456 (11%) | 304 (5.0%) |
| 70–79 | 467 (11%) | 221 (3.6%) |
| 80– | 18 (0.4%) | 22 (0.4%) |
| Sex |  |  |
| Female | 2,176 (53%) | 3,362 (55%) |
| Male | 1,958 (47%) | 2,772 (45%) |
| Living area |  |  |
| Hokkaido and Tohoku | 461 (11%) | 548 (8.9%) |
| Kanto | 1,442 (35%) | 2,285 (37%) |
| Hokuriku and Ko-shin-etsu | 250 (6.0%) | 350 (5.7%) |
| Tokai | 483 (12%) | 748 (12%) |
| Kansai | 688 (17%) | 1,175 (19%) |
| Chugoku and Shikoku | 325 (7.9%) | 488 (8.0%) |
| Kyushu and Okinawa | 485 (12%) | 540 (8.8%) |
| Academic attainment |  |  |
| Junior high school graduate | 169 (4.1%) | 229 (3.7%) |
| High school graduate | 1,449 (35%) | 2,254 (37%) |
| College graduate | 851 (21%) | 961 (16%) |
| Bachelor's degree | 1,473 (36%) | 2,405 (39%) |
| Master's or doctoral degree | 160 (3.9%) | 230 (3.7%) |
| Other | 32 (0.8%) | 55 (0.9%) |
| Marital status |  |  |
| Married | 2,187 (53%) | 2,302 (38%) |
| Never married | 1,603 (39%) | 3,621 (59%) |
| Widowed or divorced | 344 (8.3%) | 211 (3.4%) |
| Number of households |  |  |
| 1 | 949 (23%) | 1,592 (26%) |
| 2 | 1,202 (29%) | 1,268 (21%) |
| 3 | 992 (24%) | 1,438 (23%) |
| 4 | 691 (17%) | 1,216 (20%) |
| 5+ | 300 (7.3%) | 620 (10%) |
| Household equivalent income |  |  |
| Low | 362 (8.8%) | 566 (9.2%) |
| Medium | 766 (19%) | 1,215 (20%) |
| High | 2,016 (49%) | 3,050 (50%) |
| Unknown/Declined to answer | 990 (24%) | 1,303 (21%) |
| Type of employment |  |  |
| Employer | 157 (3.8%) | 253 (4.1%) |
| Self-employed | 141 (3.4%) | 122 (2.0%) |
| Regular employee | 1,511 (37%) | 2,753 (45%) |
| Non regular employee | 1,098 (27%) | 1,812 (30%) |
| Unemployed | 1,227 (30%) | 1,194 (19%) |
| ^1^Median (Q1, Q3); n (%) |  |  |

## Wave 5

| **Characteristic** | **Dropouts in wave 4** (n = 7384)^1^ | **Newly added in wave 5** (n = 6384)^1^ |
| --- | --- | --- |
| Age (median, IQR) | 35 (24, 54) | 35 (31, 39) |
| Age group |  |  |
| 16–19 | 416 (5.6%) | 51 (0.8%) |
| 20–29 | 2,657 (36%) | 1,206 (19%) |
| 30–39 | 1,068 (14%) | 3,776 (59%) |
| 40–49 | 1,033 (14%) | 589 (9.2%) |
| 50–59 | 761 (10%) | 277 (4.3%) |
| 60–69 | 652 (8.8%) | 229 (3.6%) |
| 70–79 | 726 (9.8%) | 229 (3.6%) |
| 80– | 71 (1.0%) | 27 (0.4%) |
| Sex |  |  |
| Female | 4,037 (55%) | 3,513 (55%) |
| Male | 3,347 (45%) | 2,871 (45%) |
| Living area |  |  |
| Hokkaido and Tohoku | 750 (10%) | 635 (9.9%) |
| Kanto | 2,708 (37%) | 2,317 (36%) |
| Hokuriku and Ko-shin-etsu | 419 (5.7%) | 340 (5.3%) |
| Tokai | 839 (11%) | 780 (12%) |
| Kansai | 1,278 (17%) | 1,183 (19%) |
| Chugoku and Shikoku | 616 (8.3%) | 546 (8.6%) |
| Kyushu and Okinawa | 774 (10%) | 583 (9.1%) |
| Academic attainment |  |  |
| Junior high school graduate | 298 (4.0%) | 106 (1.7%) |
| High school graduate | 2,645 (36%) | 1,271 (20%) |
| College graduate | 1,337 (18%) | 1,455 (23%) |
| Bachelor's degree | 2,771 (38%) | 3,109 (49%) |
| Master's or doctoral degree | 276 (3.7%) | 373 (5.8%) |
| Other | 57 (0.8%) | 70 (1.1%) |
| Marital status |  |  |
| Married | 3,558 (48%) | 4,546 (71%) |
| Never married | 3,289 (45%) | 1,609 (25%) |
| Widowed or divorced | 537 (7.3%) | 229 (3.6%) |
| Number of households |  |  |
| 1 | 1,801 (24%) | 968 (15%) |
| 2 | 1,968 (27%) | 1,017 (16%) |
| 3 | 1,646 (22%) | 2,090 (33%) |
| 4 | 1,322 (18%) | 1,691 (26%) |
| 5+ | 647 (8.8%) | 618 (9.7%) |
| Household equivalent income |  |  |
| Low | 663 (9.0%) | 363 (5.7%) |
| Medium | 1,457 (20%) | 920 (14%) |
| High | 3,616 (49%) | 4,018 (63%) |
| Unknown/Declined to answer | 1,648 (22%) | 1,083 (17%) |
| Type of employment |  |  |
| Employer | 261 (3.5%) | 256 (4.0%) |
| Self-employed | 207 (2.8%) | 161 (2.5%) |
| Regular employee | 2,864 (39%) | 3,034 (48%) |
| Non regular employee | 1,957 (27%) | 1,269 (20%) |
| Unemployed | 2,095 (28%) | 1,664 (26%) |
| ^1^Median (Q1, Q3); n (%) |  |  |

## Wave 6

| **Characteristic** | **Dropouts in Wave 5** (n = 5789)^1^ | **Newly added in Wave 6** (n = 7789)^1^ |
| --- | --- | --- |
| Age (median, IQR) | 37 (28, 54) | 37 (26, 52) |
| Age group |  |  |
| 16–19 | 100 (1.7%) | 754 (9.7%) |
| 20–29 | 1,529 (26%) | 2,590 (33%) |
| 30–39 | 1,623 (28%) | 781 (10%) |
| 40–49 | 778 (13%) | 1,430 (18%) |
| 50–59 | 591 (10%) | 875 (11%) |
| 60–69 | 549 (9.5%) | 899 (12%) |
| 70–79 | 547 (9.4%) | 407 (5.2%) |
| 80– | 72 (1.2%) | 53 (0.7%) |
| Sex |  |  |
| Female | 3,087 (53%) | 3,882 (50%) |
| Male | 2,702 (47%) | 3,907 (50%) |
| Living area |  |  |
| Hokkaido and Tohoku | 592 (10%) | 793 (10%) |
| Kanto | 2,091 (36%) | 2,913 (37%) |
| Hokuriku and Ko-shin-etsu | 372 (6.4%) | 418 (5.4%) |
| Tokai | 700 (12%) | 941 (12%) |
| Kansai | 968 (17%) | 1,415 (18%) |
| Chugoku and Shikoku | 468 (8.1%) | 662 (8.5%) |
| Kyushu and Okinawa | 598 (10%) | 647 (8.3%) |
| Academic attainment |  |  |
| Junior high school graduate | 93 (1.6%) | 231 (3.0%) |
| High school graduate | 1,403 (24%) | 2,085 (27%) |
| College graduate | 1,250 (22%) | 1,549 (20%) |
| Bachelor's degree | 2,657 (46%) | 3,476 (45%) |
| Master's or doctoral degree | 328 (5.7%) | 365 (4.7%) |
| Other | 58 (1.0%) | 83 (1.1%) |
| Marital status |  |  |
| Married | 3,379 (58%) | 3,768 (48%) |
| Never married | 1,998 (35%) | 3,459 (44%) |
| Widowed or divorced | 412 (7.1%) | 562 (7.2%) |
| Number of households |  |  |
| 1 | 1,212 (21%) | 2,011 (26%) |
| 2 | 1,508 (26%) | 2,109 (27%) |
| 3 | 1,474 (25%) | 1,704 (22%) |
| 4 | 1,117 (19%) | 1,353 (17%) |
| 5+ | 478 (8.3%) | 612 (7.9%) |
| Household equivalent income |  |  |
| Low | 430 (7.4%) | 808 (10%) |
| Medium | 1,052 (18%) | 1,478 (19%) |
| High | 3,102 (54%) | 3,752 (48%) |
| Unknown/Declined to answer | 1,205 (21%) | 1,751 (22%) |
| Type of employment |  |  |
| Employer | 274 (4.7%) | 265 (3.4%) |
| Self-employed | 196 (3.4%) | 252 (3.2%) |
| Regular employee | 2,425 (42%) | 3,216 (41%) |
| Non regular employee | 1,385 (24%) | 2,118 (27%) |
| Unemployed | 1,509 (26%) | 1,938 (25%) |
| y23Cresponse |  |  |
| 1 |  | 3,331 (100%) |
| Unknown |  | 4,458 |
| ^1^Median (Q1, Q3); n (%) |  |  |

## Wave 7

| **Characteristic** | **Wave 6 dropouts** (n = 9341)^1^ | **Wave 7 newly added** (n = 8341)^1^ |
| --- | --- | --- |
| Age (median, IQR) | 37 (28, 50) | 29 (25, 53) |
| Age group |  |  |
| 16–19 | 505 (5.4%) | 1,037 (12%) |
| 20–29 | 2,500 (27%) | 3,244 (39%) |
| 30–39 | 2,195 (23%) | 873 (10%) |
| 40–49 | 1,727 (18%) | 788 (9.4%) |
| 50–59 | 935 (10%) | 792 (9.5%) |
| 60–69 | 731 (7.8%) | 791 (9.5%) |
| 70–79 | 648 (6.9%) | 756 (9.1%) |
| 80– | 100 (1.1%) | 60 (0.7%) |
| Sex |  |  |
| Female | 5,186 (56%) | 4,595 (55%) |
| Male | 4,155 (44%) | 3,746 (45%) |
| Living area |  |  |
| Hokkaido and Tohoku | 990 (11%) | 968 (12%) |
| Kanto | 3,423 (37%) | 3,731 (45%) |
| Hokuriku and Ko-shin-etsu | 511 (5.5%) | 519 (6.2%) |
| Tokai | 1,113 (12%) | 1,007 (12%) |
| Kansai | 1,603 (17%) | 1,146 (14%) |
| Chugoku and Shikoku | 814 (8.7%) | 459 (5.5%) |
| Kyushu and Okinawa | 887 (9.5%) | 511 (6.1%) |
| Academic attainment |  |  |
| Junior high school graduate | 238 (2.5%) | 191 (2.3%) |
| High school graduate | 2,276 (24%) | 2,161 (26%) |
| College graduate | 2,004 (21%) | 1,601 (19%) |
| Bachelor's degree | 4,242 (45%) | 3,920 (47%) |
| Master's or doctoral degree | 481 (5.1%) | 384 (4.6%) |
| Other | 100 (1.1%) | 84 (1.0%) |
| Marital status |  |  |
| Married | 5,620 (60%) | 3,411 (41%) |
| Never married | 3,058 (33%) | 4,343 (52%) |
| Widowed or divorced | 663 (7.1%) | 587 (7.0%) |
| Number of households |  |  |
| 1 | 1,865 (20%) | 2,289 (27%) |
| 2 | 2,125 (23%) | 2,359 (28%) |
| 3 | 2,371 (25%) | 1,770 (21%) |
| 4 | 2,047 (22%) | 1,313 (16%) |
| 5+ | 933 (10.0%) | 610 (7.3%) |
| Household equivalent income |  |  |
| Low | 818 (8.8%) | 738 (8.8%) |
| Medium | 1,562 (17%) | 1,559 (19%) |
| High | 4,925 (53%) | 3,912 (47%) |
| Unknown/Declined to answer | 2,036 (22%) | 2,132 (26%) |
| Type of employment |  |  |
| Employer | 291 (3.1%) | 357 (4.3%) |
| Self-employed | 268 (2.9%) | 209 (2.5%) |
| Regular employee | 3,913 (42%) | 3,131 (38%) |
| Non regular employee | 2,322 (25%) | 2,341 (28%) |
| Unemployed | 2,547 (27%) | 2,303 (28%) |
| ^1^Median (Q1, Q3); n (%) |  |  |

## Wave 8

| **Characteristic** | **Wave 7 dropouts** (n = 5125)^1^ | **Wave 8 newly added** (n = 4125)^1^ |
| --- | --- | --- |
| Age (median, IQR) | 35 (25, 56) | 42 (33, 54) |
| Age group |  |  |
| 16–19 | 567 (11%) | 32 (0.8%) |
| 20–29 | 1,514 (30%) | 706 (17%) |
| 30–39 | 825 (16%) | 1,065 (26%) |
| 40–49 | 589 (11%) | 947 (23%) |
| 50–59 | 554 (11%) | 569 (14%) |
| 60–69 | 518 (10%) | 356 (8.6%) |
| 70–79 | 477 (9.3%) | 371 (9.0%) |
| 80– | 81 (1.6%) | 79 (1.9%) |
| Sex |  |  |
| Female | 2,711 (53%) | 2,386 (58%) |
| Male | 2,414 (47%) | 1,739 (42%) |
| Living area |  |  |
| Hokkaido and Tohoku | 542 (11%) | 443 (11%) |
| Kanto | 1,986 (39%) | 1,470 (36%) |
| Hokuriku and Ko-shin-etsu | 303 (5.9%) | 241 (5.8%) |
| Tokai | 621 (12%) | 482 (12%) |
| Kansai | 814 (16%) | 743 (18%) |
| Chugoku and Shikoku | 419 (8.2%) | 340 (8.2%) |
| Kyushu and Okinawa | 440 (8.6%) | 406 (9.8%) |
| Academic attainment |  |  |
| Junior high school graduate | 115 (2.2%) | 71 (1.7%) |
| High school graduate | 1,363 (27%) | 1,031 (25%) |
| College graduate | 1,019 (20%) | 921 (22%) |
| Bachelor's degree | 2,325 (45%) | 1,860 (45%) |
| Master's or doctoral degree | 241 (4.7%) | 204 (4.9%) |
| Other | 62 (1.2%) | 38 (0.9%) |
| Marital status |  |  |
| Married | 2,316 (45%) | 2,772 (67%) |
| Never married | 2,415 (47%) | 1,023 (25%) |
| Widowed or divorced | 394 (7.7%) | 330 (8.0%) |
| Number of households |  |  |
| 1 | 1,359 (27%) | 685 (17%) |
| 2 | 1,438 (28%) | 942 (23%) |
| 3 | 1,089 (21%) | 1,019 (25%) |
| 4 | 851 (17%) | 1,044 (25%) |
| 5+ | 388 (7.6%) | 435 (11%) |
| Household equivalent income |  |  |
| Low | 489 (9.5%) | 321 (7.8%) |
| Medium | 929 (18%) | 621 (15%) |
| High | 2,397 (47%) | 2,206 (53%) |
| Unknown/Declined to answer | 1,310 (26%) | 977 (24%) |
| Type of employment |  |  |
| Employer | 242 (4.7%) | 138 (3.3%) |
| Self-employed | 135 (2.6%) | 123 (3.0%) |
| Regular employee | 1,874 (37%) | 1,748 (42%) |
| Non regular employee | 1,402 (27%) | 1,015 (25%) |
| Unemployed | 1,472 (29%) | 1,101 (27%) |
| ^1^Median (Q1, Q3); n (%) |  |  |

# Supplementary Method

Previous studies have suggested that the adjusted estimates using inverse probability weighting (IPW) obtained from a propensity score (calculated by logistic regression models using basic demographic and socio-economic factors such as education and housing tenure) from an internet-based convenience sample provide similar estimates of parameters, or at least reduced the differences compared to population-based estimates. Therefore, IPW-adjusted estimates are also presented in this study. To correct for the selectivity of internet-based samples, we used a population-based sample that is representative of the Japanese population from the Comprehensive Survey of Living Conditions of People on Health and Welfare (CSLCPHW). Data from two surveys (internet survey and CSLCPHW) were pooled (combined) and used for a logistic regression model with all the above-mentioned covariates to estimate the probability of ‘being a respondent in an internet survey’, i.e. propensity score. Using sex and age group stratifications (sex × age groups = 14 strata), we calculated the propensity score separately for each stratum. Data from the 2016 CSLCPHW were used as 2019 CSLCPHW was not yet available at the time of analysis.
